# Belief about the future possibility of national aging security system and its association with mortality

**DOI:** 10.1371/journal.pone.0212282

**Published:** 2019-02-14

**Authors:** Doukyoung Chon, Ki Bong Yoo, Jae-Hyun Kim

**Affiliations:** 1 Department of Preventive Medicine and Public Health, Ajou University School of Medicine, Suwon, Republic of Korea; 2 Institute on Aging, Ajou University Medical Center, Suwon, Republic of Korea; 3 College of Health Sciences, Yonsei University, Wonju, Korea; 4 Department of Health Administration, College of Health Science, Dankook University, Cheonan, Korea; 5 Institute of Health Promotion and Policy, Dankook University, Cheonan, Korea; Indiana University Purdue University at Indianapolis, UNITED STATES

## Abstract

In line with well-known subjective measures of health, such as self-rated health and subjective life expectancy, an individual’s belief about future security provided by the government could also be an important factor affecting his life expectancy. The aim of this study was to use the response of the elderly Korean population in regards to the National Aging Security System (NASS), and assess its association with the risk of mortality even with SRH included in the analysis. Data from the Korean Longitudinal Study of Ageing (KLoSA) from 2006 to 2016 were assessed using longitudinal data analysis and 10,254 research subjects were included at baseline in 2006. To analyze the association between belief about future possibility of NASS and all-cause mortality, Cox proportional hazards model was used. In terms of the future possibility of NASS, people who thought more negatively displayed greater risk of mortality at the end of the follow-up. With the Positive group as reference: Moderate group showed a 18% increase (HR = 1.178, 95% CI: 1.022, 1.357), and Negative groups showed a 19% increase (HR = 1.192, 95% CI: 1.043, 1.362). The results of our study showed that people’s belief regarding future security could be associated with mortality rates. Our finding is important, because it provides additional support to the importance of considering subjective measures of health in epidemiological research. Furthermore, the findings of our research could be useful in terms of future policy making.

## Introduction

Increasing number of studies are considering subjective measures of health in addition to the traditional measures of health in association with mortality [1, 2]. This is a newer trend in this area of research, because in the past, researchers often used more objective measures of health such as smoking status, blood pressure level, and history of congestive heart failure [3]. The reason for the use of subjective measures of health can be seen in studies such as the one by Strawbridge and Wallhagen [4] that demonstrated the independent effect of self-rated health (SRH) on the risk of mortality. Likewise, subjective measures concern the belief about one’s own health or state of well-being, which could concern the past, the present or the future.

SRH is mainly used as a measure of current health state, and the predictability of SRH on the risk of mortality was demonstrated throughout studies in the past. For example, Fried et al. [3] assessed various risk factors in terms of mortality, and worse SRH was associated with higher risk of mortality in the unadjusted model as well as the fully adjusted model. Also, Idler et al. [5] found similar predictability of SRH on the risk of mortality, but it was only observed in participants suffering from circulatory system disease. Therefore, effects of SRH on different groups of people need to be considered.

Another subjective health measure that is often used in research is subjective life expectancy (SLE). SLE is an individual’s subjective opinion of remaining length of life, and it has shown to be associated with mortality risk [6], even with SRH in consideration [2]. Hence, SRH and SLE both affect mortality and are conceptually similar, but they also show independent effects on mortality. Several factors, such as emotional support [7], heath status [8], and feeling secure about one’s future [9] could influence an individual’s SLE. Furthermore, people’s sense of future security could be affected by their level of confidence in regards to their government.

Previously, reduction in mortality in relation to improvements in government provided social services was observed. For example, the Social Security in the United States [10] and The Estrategia de Saude da Familia in Brazil [11] were both associated with reduced mortality. Similar government provided social security systems, such as the Universal health insurance as well as the Medical Aid program provided by the government, exist in Korea [12]. These programs are important, because Korea has one of the fastest growing elderly population as well as the highest elderly poverty rate among OECD countries [13]. Considering previous studies [10, 11] these government programs could improve the health status and reduce the mortality rate of Koreans, but how confident people feel about those programs is a different matter. We hypothesized that people’s level of confidence in the National Aging Security System (NASS) could be a good indicator of how secure people felt about their future, and aimed to study the association between the response of the elderly Korean population in regards to the National Aging Security System (NASS) and mortality.

## Methods

### Study sample

Data were obtained from the 2006, 2008, 2010, 2012, 2014 and 2016 waves of the Korean Longitudinal Study of Ageing (KLoSA). Sampling was conducted by sorting the population surveyed in a given area and 15 residential type according to the order of the administrative codes, and then extracting the assigned number by applying a systematic extraction method (the multistage and stratified randomized sampling method). As per the KLoSA study protocol, trained surveyors collected informed consents from participants and conducted face-to-face interviews using a computer-assisted personal interviewing program.

In the first baseline survey in 2006, 10,254 individuals in 6,171 households (1.7 per household) were interviewed. There were 292 individuals with cancer. The second survey, in 2008, followed up with 8,675 subjects, who represented 86.6% of the original panel. The third survey, in 2010, followed up with 8,229 subjects, who represented 81.7% of the original panel, the fourth survey, in 2012, followed up with 7,813 subjects, who represented 80.1% of the original panel and the fifth survey, in 2014, followed up with 8,387 subjects (including 920 new participated sample), who represented 80.4% of the original panel. The sixth survey, in 2016, followed up with 9,913 subjects (including 878 new participated sample), who represented 79.6% of the original panel.

### Independent variables

Belief about the future possibility of National Aging Security System (NASS) measures a continuum of subjective probabilities using responses to a statement “I believe that the government will provide security in my later years.” The response to the question ranges from 0 to 100, where 0 means that you think there is absolutely no chance, and 100 meaning that you think the event is absolutely sure to happen. Belief about the future possibility of NASS was divided into three groups: negative, moderate and positive using SAS Rank function.

### Control variables

From the first wave, covariates were collected: age (45–49, 50–59, 60–69, ≥70), gender (male and female), education (elementary, middle, high school, and ≥college), residential region (Urban: administrative divisions of a city: Seoul, Daejeon, Daegu, Busan, Incheon, Kwangju, or Ulsan, Rural: not classified as administrative of a city), income level (<100, 100–199, 200–299, and ≥300 in thousand won), smoking status (non-smoker, former smoker and smoker), alcohol consumption (nothing, former drinker and drinker), Marital status (married’ and single which includes ‘separated’, ‘divorced’, ‘widowed or disappeared’, and ‘never married’). The type of medical insurance (National Health Insurance beneficiary or Medical Aid beneficiary), free national health screening (Yes or No), self-rated health (very good, good, fair, poor, very poor), government pension (Yes or No), number of hospitalization before 1year, number of outpatient use before 1 year and comorbidity of hypertension, diabetes, cancer, chronic obstructive pulmonary disease, liver disease, heart disease, cerebrovascular diseases and mental illness (0, 1, and ≥2). Social engagement was measured using five variables. Briefly, (1) frequency of contacts of friends (4: every day, 3: once a month–two or three per week, 2: once a year–five or six a year or almost nothing); (2) frequency of contacts within a mutual benevolence group meeting (4: every day, 3: once a month–two or three per week, 2: once a year–five or six a year or almost nothing); (3) frequency of attendance at leisure, culture, and sports activities (4: every day, 3: once a month–two or three per week, 2: once a year–five or six a year or almost nothing**)**; (4) frequency of religious attendance (4: every day, 3: once a month–two or three per week, 2: once a year–five or six a year or almost nothing); and (5) frequency of contacts at an alumni meeting or hometown alumni and clan gathering (4: every day, 3: once a month–two or three per week, 2: once a year–five or six a year or almost nothing). The variables were summed, with totals ranging from 4 to 20. Social engagement was ranked from lowest to highest, and four groups were analyzed using the SAS rank function.

### Dependent variables

#### All-cause mortality

All-cause mortality during the time interval from year 2006 to the end of follow-up was the main outcome of the study. Death over a maximum follow-up period of 10 years was determined by death certificates and coroner’s report.

### Analytical approach and statistics

First, we employed descriptive statistics to describe general characteristics of the sample through chi-square test. Then, we used the Cox proportional hazards model to investigate the associations between belief about the future possibility of NASS and all-cause mortality. For all analyses, the criterion for statistical significance was p <0.05, two-tailed. All analyses were conducted using the SAS statistical software package, version 9.4 (SAS Institute Inc., Cary, NC, USA).

## Results

### Sample characteristics

Baseline characteristics of participants are shown in Table 1. Of the total of 9,374 participants, there were 1,347 (14.4%) mortality at the end of the follow-up. In terms of the future possibility of NASS, there were 366 (11.4%) mortality in the Positive group, 412 (14.6%) mortality in the Moderate group, and 569 (17.0%) mortality in the Negative group. In terms of SRH, there were 251 (39.2%) mortality in the Very insufficient group, 504 (22.6%) mortality in the Insufficient group, 356 (12.2%) mortality in the Normal group, 218 (6.7%) mortality in the Sufficient group, and 18 (5.6%) mortality in the Very sufficient group.

**Table 1:** General characteristics of participants at baseline.

	Total		Death				P-value
			No		Yes		
	N/mean	%/SD	N/mean	%/SD	N/mean	%/SD	
**Future possibility of national aging security system**							< .0001
**Negative**	3,349	35.7	2,780	83.0	569	17.0	
**Moderate**	2,825	30.1	2,413	85.4	412	14.6	
**Positive**	3,200	34.1	2,834	88.6	366	11.4	
**Age**							< .0001
**45–54**	1,749	18.7	1,705	97.5	44	2.5	
**55–64**	2,829	30.2	2,691	95.1	138	4.9	
**65–74**	2,358	25.2	2,081	88.3	277	11.8	
**≥74**	2,438	26.0	1,550	63.6	888	36.4	
**Gender**							< .0001
**Male**	3,900	41.6	3,216	82.5	684	17.5	
**Female**	5,474	58.4	4,811	87.9	663	12.1	
**Education**							< .0001
**≤Elementary**	4,361	46.5	3,410	78.2	951	21.8	
**Middle school**	1,538	16.4	1,392	90.5	146	9.5	
**High school**	2,498	26.7	2,321	92.9	177	7.1	
**≥College**	977	10.4	904	92.5	73	7.5	
**Residential region**							< .0001
**Urban**	6,121	65.3	5,336	87.2	785	12.8	
**Rural**	3,253	34.7	2,691	82.7	562	17.3	
Income level (thousand won)*							< .0001
**<100**	8,142	86.9	6,843	84.1	1,299	16.0	
**100–199**	582	6.2	557	95.7	25	4.3	
**200–299**	289	3.1	276	95.5	13	4.5	
**≥300**	361	3.9	351	97.2	10	2.8	
**Smoking status**							< .0001
**Non-smoker**	6,772	72.2	5,921	87.4	851	12.6	
**Former smoker**	845	9.0	647	76.6	198	23.4	
**Smoker**	1,757	18.7	1,459	83.0	298	17.0	
**Alcohol consumption**							< .0001
**Nothing**	3,488	37.2	3,074	88.1	414	11.9	
**Former drinker**	583	6.2	417	71.5	166	28.5	
**Drinker**	5,303	56.6	4,536	85.5	767	14.5	
**Marital status**							< .0001
**Married**	7,307	78.0	6,495	88.9	812	11.1	
**Single (including separated, divorced)**	2,067	22.1	1,532	74.1	535	25.9	
Social engagement**							< .0001
**Low**	2,450	26.1	1,891	77.2	559	22.8	
**Middle low**	1,049	11.2	875	83.4	174	16.6	
**Middle**	2,434	26.0	2,155	88.5	279	11.5	
**Middle high**	1,268	13.5	1,136	89.6	132	10.4	
**High**	2,173	23.2	1,970	90.7	203	9.3	
**National health insurance**							< .0001
**National health insurance beneficiary**	8,782	93.7	7,581	86.3	1,201	13.7	
**Medical aid beneficiary**	592	6.3	446	75.3	146	24.7	
**Private health insurance**							< .0001
**Yes**	3,049	32.5	2,933	96.2	116	3.8	
**No**	6,325	67.5	5,094	80.5	1,231	19.5	
**Free national health screening**							< .0001
**Yes**	4,197	44.8	3,708	88.4	489	11.7	
**No**	5,177	55.2	4,319	83.4	858	16.6	
**Self-rated health**							< .0001
**Very sufficient**	323	3.5	305	94.4	18	5.6	
**Sufficient**	3,256	34.7	3,038	93.3	218	6.7	
**Normal**	2,924	31.2	2,568	87.8	356	12.2	
**Insufficient**	2,231	23.8	1,727	77.4	504	22.6	
**Very insufficient**	640	6.8	389	60.8	251	39.2	
**Government pension**							< .0001
**Yes**	2,041	21.8	1,963	96.2	78	3.8	
**No**	7,333	78.2	6,064	82.7	1,269	17.3	
**Number of hospitalization before 1 year**	0	0.7	0	0.6	0	1.0	
**Number of outpatient use before 1 year**	6	15.8	6	15.3	8	18.2	
Number of chronic diseases***							< .0001
**0**	4,980	53.1	4,495	90.3	485	9.7	
**1**	2,681	28.6	2,239	83.5	442	16.5	
**≥2**	1,713	18.3	1,293	75.5	420	24.5	
**Total**	9,374	100.0	8,027	85.6	1,347	14.4	

*Unit: 1,000KRW = 1$

**Social engagement was measured in five variables. (1) frequency of contacts in domains of friends (2) frequency of contacts in mutual benevolence group meeting (3) frequency of attendance in leisure, culture and sports activities (4) frequency of religious attendance (5) frequency of contacts in alumni meeting, hometown alumni and clan gathering

***Hypertension, diabetes, cancer, chronic obstructive pulmonary disease, liver disease, heart disease, cerebrovascular diseases, mental illness and arthritis or rheumatoid arthritis

### Adjusted effects of future possibility of NASS on death

In terms of the future possibility of NASS, people who thought more negatively displayed greater risk of mortality at the end of follow-up. With the Positive group as reference: Moderate group showed an 18% increase (HR = 1.178, 95% CI: 1.022, 1.357), and Negative groups showed a 19% increase (HR = 1.192, 95% CI: 1.043, 1.362). Kaplan-Meier curve for all-cause mortality is shown in S1 Fig. SRH was also significantly associated with the risk of mortality, where as participants felt more poorly about their health at baseline, their risk of mortality increased at the end of follow-up. However, with the Very good group as reference, only the Very poor group (HR = 2.288, 95% CI: 1.395, 3.753) showed a significant increase in the risk of mortality (Table 2).

**Table 2:** Adjusted effect of future possibility of national aging security system on death.

	Death			
	HR	95% CI		P-value
**Future possibility of national aging security system**				
**Negative**	1.192	1.043	1.362	0.010
**Moderate**	1.178	1.022	1.357	0.024
**Positive**	1.000			
**Age**				
**45–54**	1.000			
**55–64**	1.649	1.169	2.325	0.004
**65–74**	2.722	1.935	3.830	< .0001
**≥74**	7.095	5.049	9.970	< .0001
**Gender**				
**Male**	2.324	1.977	2.731	< .0001
**Female**	1.000			
**Education**				
**≤Elementary**	1.292	0.998	1.673	0.051
**Middle school**	1.097	0.822	1.464	0.529
**High school**	1.053	0.798	1.389	0.714
**≥College**	1.000			
**Residential region**				
**Urban**	1.000			
**Rural**	1.264	1.129	1.415	< .0001
Income level (thousand won)*				
**<100**	1.000			
**100–199**	0.666	0.442	1.005	0.053
**200–299**	0.874	0.496	1.539	0.640
**≥300**	0.650	0.341	1.239	0.191
**Smoking status**				
**Non-smoker**	1.000			
**Former smoker**	1.334	1.108	1.607	0.002
**Smoker**	1.328	1.131	1.560	0.001
**Alcohol consumption**				
**Nothing**	1.000			
**Former drinker**	1.099	0.909	1.328	0.330
**Drinker**	1.113	0.961	1.288	0.153
**Marital status**				
**Married**	1.000			
**Single (including separated, divorced)**	1.632	1.432	1.860	< .0001
Social engagement**				
**Low**	1.563	1.323	1.846	< .0001
**Middle low**	1.188	0.966	1.462	0.103
**Middle**	1.189	0.990	1.428	0.064
**Middle high**	1.167	0.935	1.455	0.172
**High**	1.000			
**National health insurance**				
**National health insurance beneficiary**	1.000			
**Medical aid beneficiary**	0.968	0.810	1.156	0.719
**Private health insurance**				
**Yes**	1.000			
**No**	1.398	1.128	1.734	0.002
**Free national health screening**				
**Yes**	1.000			
**No**	1.293	1.154	1.450	< .0001
**Self-rated health**				
**Very sufficient**	1.000			
**Sufficient**	0.940	0.580	1.523	0.802
**Normal**	1.107	0.686	1.787	0.677
**Insufficient**	1.526	0.943	2.469	0.086
**Very insufficient**	2.288	1.395	3.753	0.001
**Government pension**				
**Yes**	1.000			
**No**	1.431	1.103	1.857	0.007
**Number of hospitalization before 1 year**	1.092	1.037	1.149	0.001
**Number of outpatient use before 1 year**	0.997	0.994	1.000	0.088
Number of chronic diseases***				
**0**	1.000			
**1**	0.990	0.864	1.133	0.882
**≥2**	1.124	0.969	1.305	0.122

*Unit: 1,000KRW = 1$

**Social engagement was measured in five variables. (1) frequency of contacts in domains of friends (2) frequency of contacts in mutual benevolence group meeting (3) frequency of attendance in leisure, culture and sports activities (4) frequency of religious attendance (5) frequency of contacts in alumni meeting, hometown alumni and clan gathering

***Hypertension, diabetes, cancer, chronic obstructive pulmonary disease, liver disease, heart disease, cerebrovascular diseases, mental illness and arthritis or rheumatoid arthritis

### Stratification analysis of future possibility of NASS

Final analysis of the study included stratification of variables, which were statistically significant in the previous model. In terms of gender, future possibility of NASS was no longer significantly associated with the risk of mortality for males. Significant association remained in the Female group, but it was only significant in the Negative group (HR = 1.268, 95% CI: 1.047, 1.536) compared to the Positive group. The future possibility of NASS was not significantly associated with the risk of mortality in the group with private health insurance, but for those without it, the risk of mortality was higher in the Moderate group (HR = 1.175, 95% CI: 1.011, 1.365) as well as the Negative group (HR = 1.205, 95% CI: 1.048, 1.386) compared to the Positive group. Also, the future possibility of NASS was not significantly associated with the risk of mortality for people with free national health screening, but for those without it, the association was significant for both the Moderate group (HR = 1.297, 95% CI: 1.081, 1.556), and the Negative group (HR = 1.247, 95% CI: 1.051, 1.478). Finally, significant association between the future possibility of NASS and the risk of mortality was not observed in those under the age of 60, but the risk of mortality was significantly higher in the Negative group compared to the Positive group for those aged 60 years and older (HR = 1.214, 95% CI: 1.051, 1.402), as well as those in a more specified age group, 60–79 years old, (HR = 1.203, 95% CI: 1.01, 1.433)(Table 3).

**Table 3:** Stratification analysis of future possibility of national aging security system on death.

		Death			
Future possibility of national aging security system		HR	95% CI		P-value
Gender*	**Male**				
	**Negative**	1.100	0.911	1.328	0.322
	**Moderate**	1.215	0.997	1.480	0.054
	**Positive**	1.000			
	**Female**				
	**Negative**	1.268	1.047	1.536	0.015
	**Moderate**	1.129	0.918	1.389	0.249
	**Positive**	1.000			
Private health insurance*	**Yes**				
	**Negative**	0.963	0.605	1.533	0.874
	**Moderate**	1.186	0.757	1.859	0.457
	**Positive**	1.000			
	**No**				
	**Negative**	1.205	1.048	1.386	0.009
	**Moderate**	1.175	1.011	1.365	0.035
	**Positive**	1.000			
Free national health screening*	**Yes**				
	**Negative**	1.111	0.893	1.383	0.344
	**Moderate**	1.044	0.829	1.314	0.716
	**Positive**	1.000			
	**No**				
	**Negative**	1.247	1.051	1.478	0.011
	**Moderate**	1.297	1.081	1.556	0.005
	**Positive**	1.000			
Age*	**<64**				
	**Negative**	1.069	0.736	1.551	0.726
	**Moderate**	1.234	0.865	1.761	0.247
	**Positive**	1.000			
	**≥65**				
	**Negative**	1.214	1.051	1.402	0.008
	**Moderate**	1.149	0.984	1.342	0.080
	**Positive**	1.000			

*Adjusted for age. Gender, education, residential region, income level, smoking status, alcohol consumption, marital status, social engagement, national health insurance, free national health screening, self-rated health, government pension, number of hospitalization, number of outpatient use and number of chronic diseases

## Discussion

In this study considering the factors that could affect the risk of mortality, people’s thoughts on their future security was significantly associated with the risk of mortality. The risk of mortality was 19% higher for people in the Negative group, in terms of the future possibility of NASS, compared to the Positive group, and this could be viewed in similar regards as feeling hopeless. Everson et al. [14] surveyed people’s level of hopelessness in association with the risk of mortality and found a significant association. Likewise, it is possible that when people do not sense a promising future security, their level of hopelessness increases, and consequently increases the risk of mortality.

In our stratified analysis, thoughts on the future possibility of NASS was significantly associated with the risk of mortality for women, but not for men. The possible reason for this association could be due to the differences in health behaviors between men and women, when they feel similarly about the future. In a past study by Scott-Sheldon et al. [15], men and women, who felt similarly about their life expectancy, displayed different health behaviors. In terms of both private health insurance and free national health screening, elders without these benefits were more significantly affected by their belief about future possibility of NASS. This could also be associated with the fact that increased number of concurrent problems worsens the perception of one’s future [9]. Furthermore, association between NASS and mortality was only significant in elders aged 60 years and older.

Until now, various studies in this field of research have used SRH [16] and SLE [17] as subjective measures of health. Our study is similar to previous studies in the sense that it measures subjective measures, but it also includes a new concept, which is related to how positively people feel about their future. In the past, SLE was often used as a futuristic subjective perception of oneself[18]. Because, having a sense of security about one’s own future influences people’s SLE [9], ratings on the future possibility of NASS was used in our study.

Our findings are important, because it provides indications to the importance of implementation of a better NASS as well as improved subjective ratings about oneself,. Benefits of providing better universal security system have been shown in previous studies [19, 20], and as important as it is to implement better systems at the government level, it could be equally important to educate people and provide information, so people understand the benefits provided by the government and feel a stronger sense of security regarding their future. Therefore, implementing a better system to elevate people’s well-being could improve the risk of mortality on its own, as well as by providing a more positive sense of future security.

The strengths of the current study are the use of a population-based representative sample with a 10-year follow-up. Also, the study was designed to analyze the prospective effects of SRH and the belief about the future possibility of NASS on the risk of mortality 10 years later. Using a more specific measure of people’s subjective ratings was also beneficial in the sense that it narrowed down where our attention needs to be focused on.

Although, this study provides various strengths, certain limitations also need to be mentioned. First, the independent variable of the study was based on self-reports, which could sometimes be less accurate, compared to more objective measures. However, number of studies have demonstrated how subjective measures sometimes provide better explanation of people’s status compared to more objective measures [8, 21], making this problem less significant. Second, there could have been other confounding factors that were not accounted for in our study. However, given the nature of a secondary data, included variables were limited. Lastly, the cause of mortality was not specified in our study, and considering this factor in future studies could benefit this line of work.

This study provided additional results to the line of work considering subjective measures of health. Additionally, our study provided a new subjective rating that could assist in future policy making. Future studies considering better national security system as well as the level of confidence people have about the system would be beneficial in regards to the risk of mortality, even possibly the quality of life itself.

## Supporting information

S1 Fig Kaplan-meier curve for association between future possibility of national aging security system and all-cause mortality. (TIF)

S1 File “Data06.sas7bdat”. (SAS7BDAT)
