# Correction: Belief about the future possibility of national aging security system and its association with mortality

**DOI:** 10.1371/journal.pone.0303116

**Published:** 2024-05-01

**Authors:** The PLOS ONE Editors

There are errors in the Author Contributions of the second author, Ki Bong Yoo. The correct contributions are: Formal analysis, Investigation, Methodology, Software, Validation, and Writing–review & editing
