# Supplementary figures and images for: Belief about the future possibility of national aging security system and its association with mortality
Source: PLoS One. 2019 Feb 14;14(2):e0212282. doi: 10.1371/journal.pone.0212282 (PMC6375624; doi:10.1371/journal.pone.0212282)

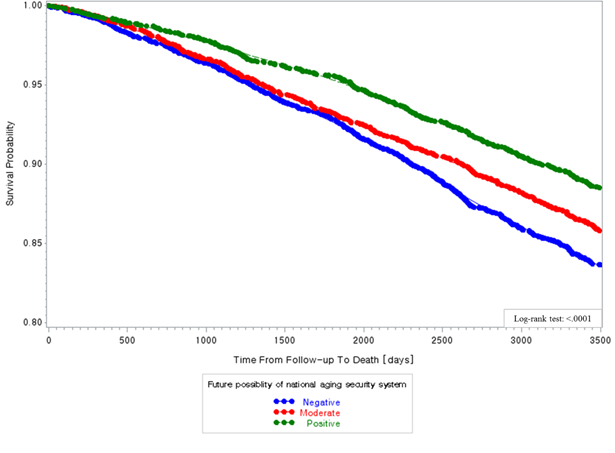

Supplement: S1 Fig — (TIF) [file pone.0212282.s001.tif]
